# Supplementary material for: Photothermia at the nanoscale induces ferroptosis via nanoparticle degradation
Source: Nat Commun. 2023 Aug 2;14:4637. doi: 10.1038/s41467-023-40258-1 (PMC10397343; doi:10.1038/s41467-023-40258-1)
Supplement: Supplementary file 1 — supplementary information [file 41467_2023_40258_MOESM1_ESM.pdf]

SUPPLEMENTARY INFORMATION

Photothermia at the nanoscale induces ferroptosis via nanoparticle degradation

Alexandre Fromain[1+], Jose Efrain Perez[1+], Aurore Van de Walle[1], Yoann Lalatonne[2], and Claire Wilhelm[1*]

1. Laboratoire Physico Chimie Curie, PCC, CNRS UMR168, Institut Curie, Sorbonne University, PSL University, 75005 Paris, France. 2. Université Sorbonne Paris Nord, Université Paris Cité, Laboratory for Vascular Translational Science, LVTS, INSERM, UMR 1148, Bobigny F-93017, France. Département de Biophysique et de Médecine Nucléaire, Assistance Publique-Hôpitaux de Paris, Hôpital Avicenne F- 93009, Bobigny, France.

[+] Equal contribution * claire.wilhelm@cnrs.fr

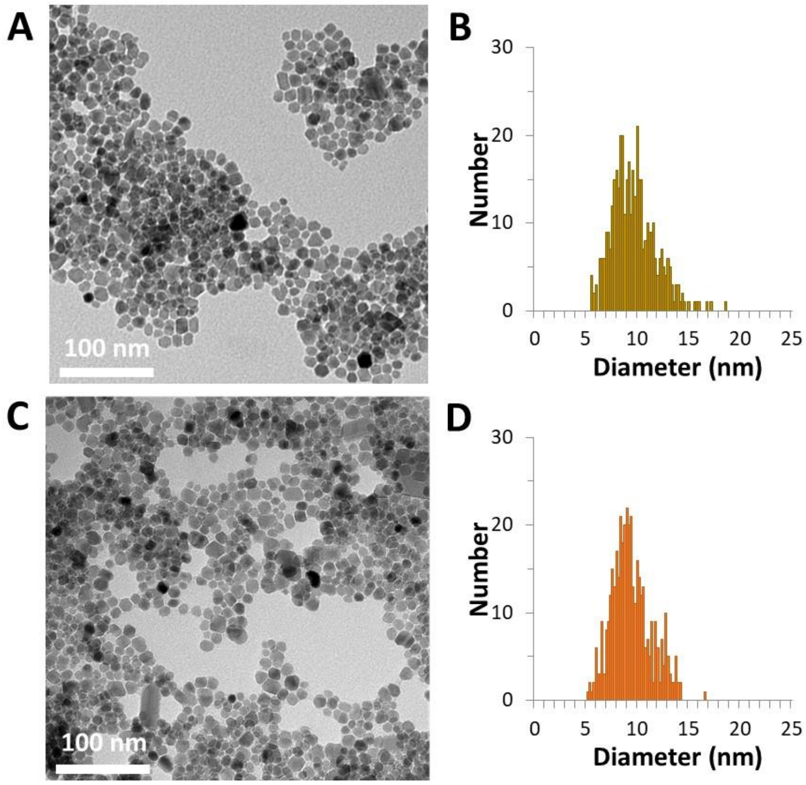

Supplementary Figure 1: Transmission Electron microscopy imaging of citrate-coated iron oxide nanoparticles (A) and associated size distribution (B), with the same analysis for the oxidized nanoparticles (C, D); The mean diameters for both nanoparticles are similar, at 9.6 ± 2.1 nm and 9.4 ± 2 nm for non-oxidized and oxidized nanoparticles, respectively.

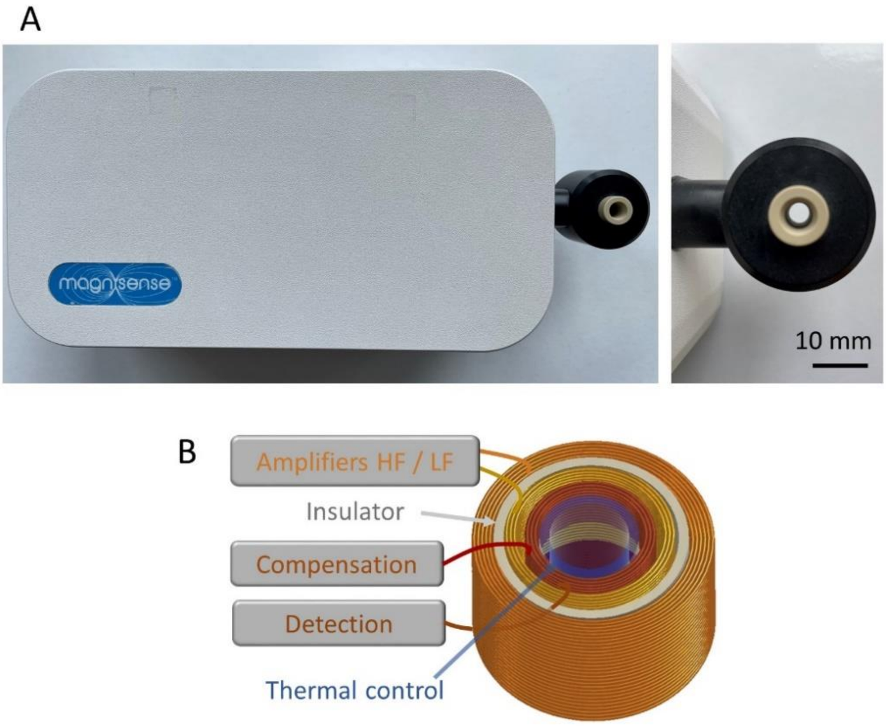

Supplementary Figure 2: Images of the magnetic sensor device (A) and scheme describing the arrangement of coils to perform the frequency-dependent measure (B). HF = high frequency; LF = low frequency. In brief, this detection method is based on the nonlinear magnetization of magnetic sample, and consists in measuring this nonlinear response upon exposure to a two-frequency alternating magnetic field. The two-frequency fields are generated by two independent coils, in a far range from 100 Hz to 100 kHz, and with different magnetic field amplitudes of 20 mT and 1 mT, respectively. The concept is to magnetize the nanoparticles with a low frequency field, and then switch this magnetization sinusoidally at high frequency so that the sensing coil voltage will be modulated by both frequencies. Combinatorial Fourier transform analysis finally provides the third derivative of the sample magnetization around zero field and room temperature. The most powerful aspect of this detection approach is that the combinatorial measurement removes all background noise.

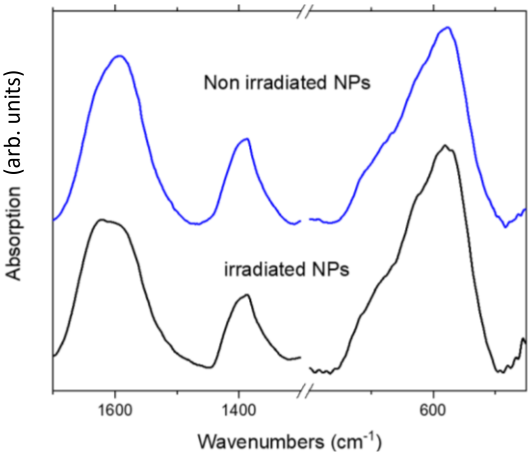

Supplementary Figure 3: Fourier Transform Infrared (FTIR, Nicolet 380, Thermo Fisher Scientific) spectra of citrate-coated non-oxidized (NP) before (blue curve) and after NIR laser irradiation (black curve). Laser irradiation in water does not affect neither the Fe-O vibration band within the 800-450 cm^{-1} region nor C-O stretches within the 1700–1300 cm^{-1} region. This is respectively correlated to unchanged coating surface and crystallinity.

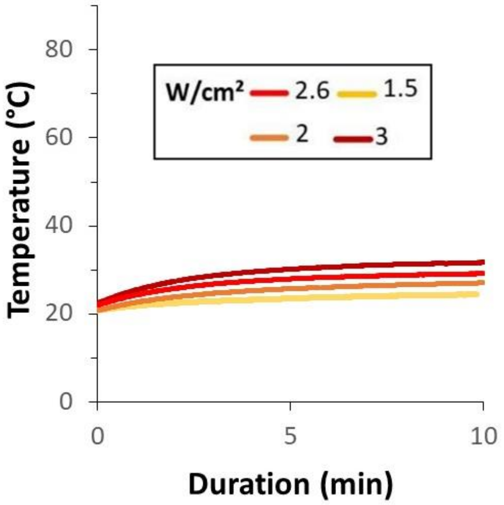

Supplementary Figure 4: Temperature increase of a solution of ferrous ascorbate at [Fe] = 2 mM in water exposed to increasing 808 nm laser power densities under the same experimental settings of the heating recorded with nanoparticles presented in Figure 2B. The temperature increases at 1.5, 2, 2.6, and 3 W/cm² were 3, 5, 7 and 9°C, compared to 22, 34, 44 and 58°C for ferrous ascorbate and iron oxide nanoparticles, respectively. Overall, Fe^{2+} provide less than 15% of the heating efficiency of nanoparticles.

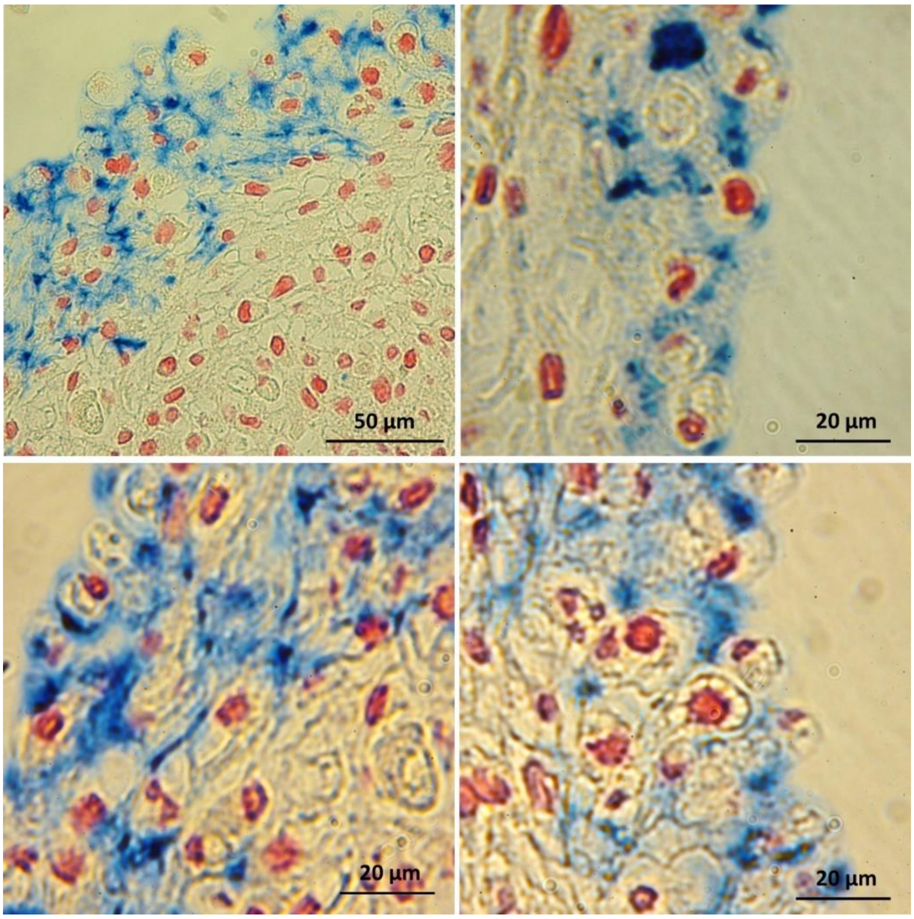

Supplementary Figure 5: Prussian blue staining for early endosomes condition (30 min internalization).

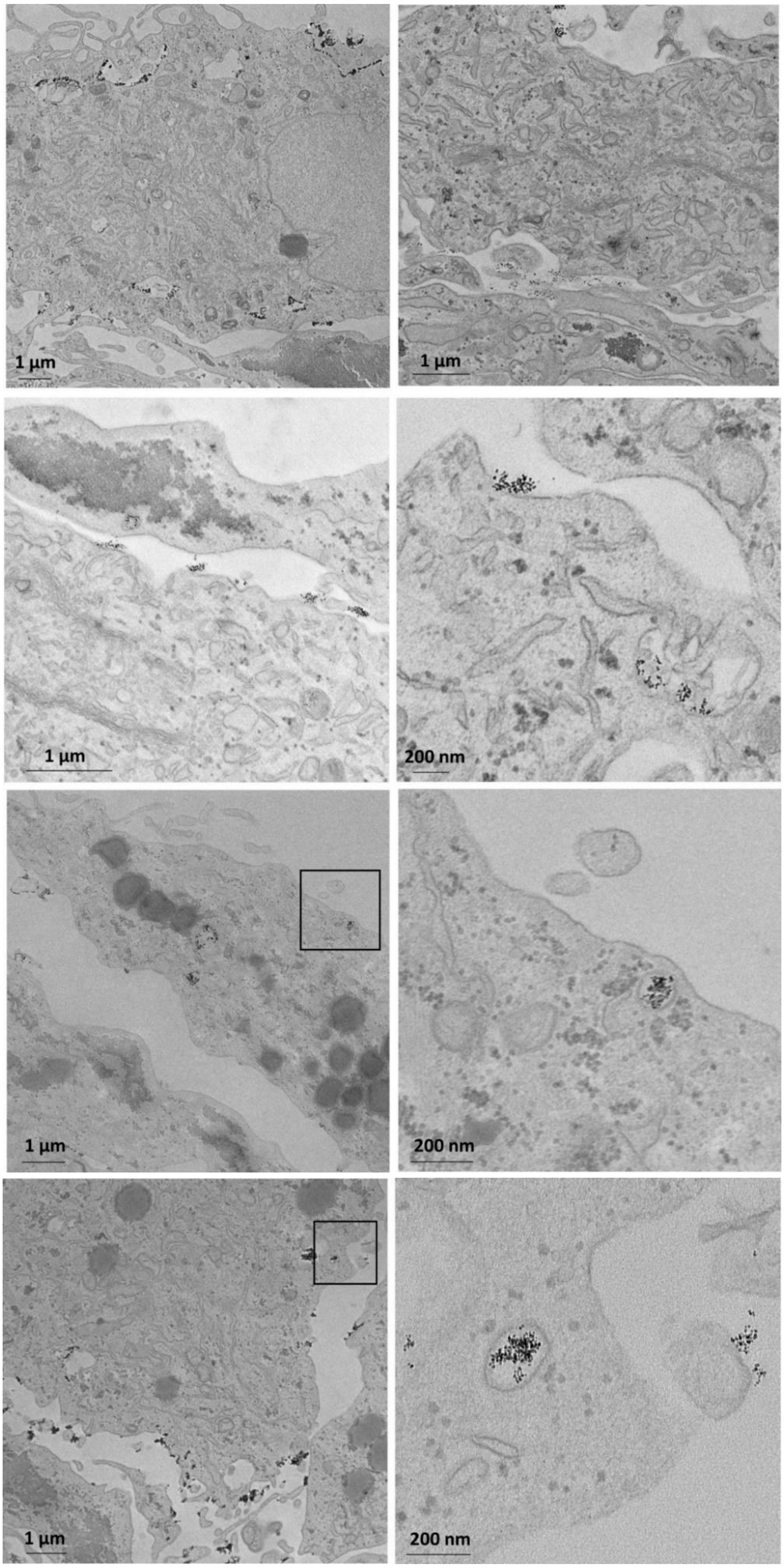

Supplementary Figure 6: TEM images of spheroids after 30 min internalization of the nanoparticles.

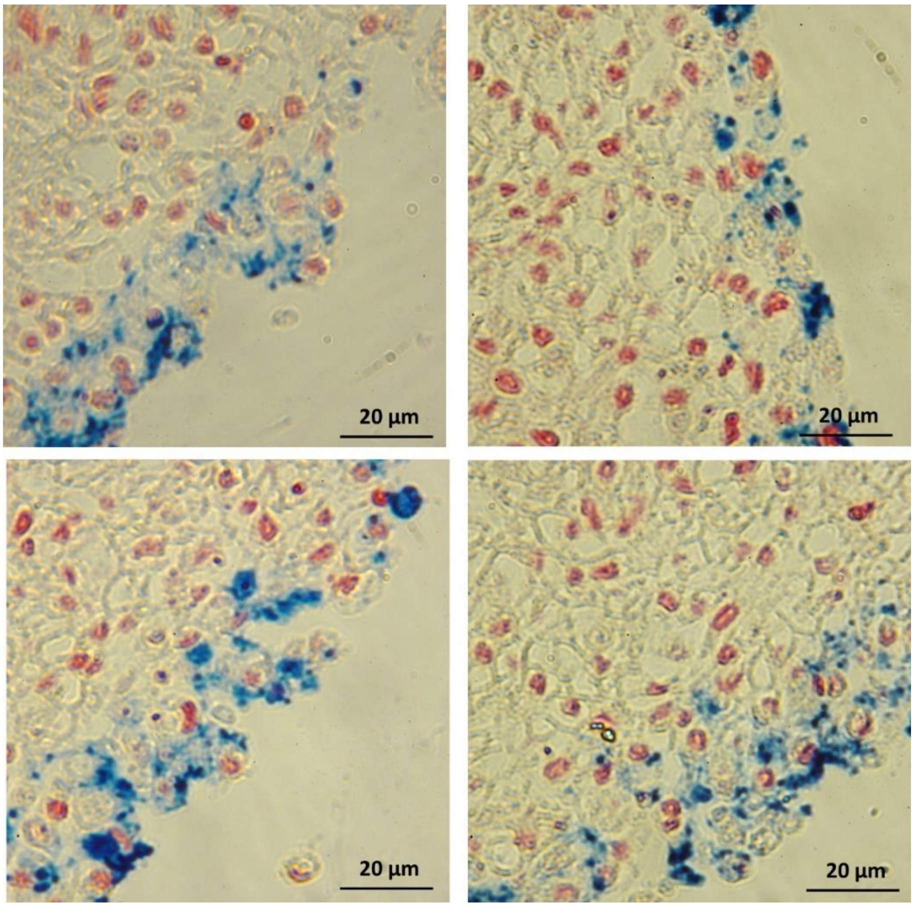

Supplementary Figure 7: Prussian blue staining for late endosomes condition (4 hours internalization).

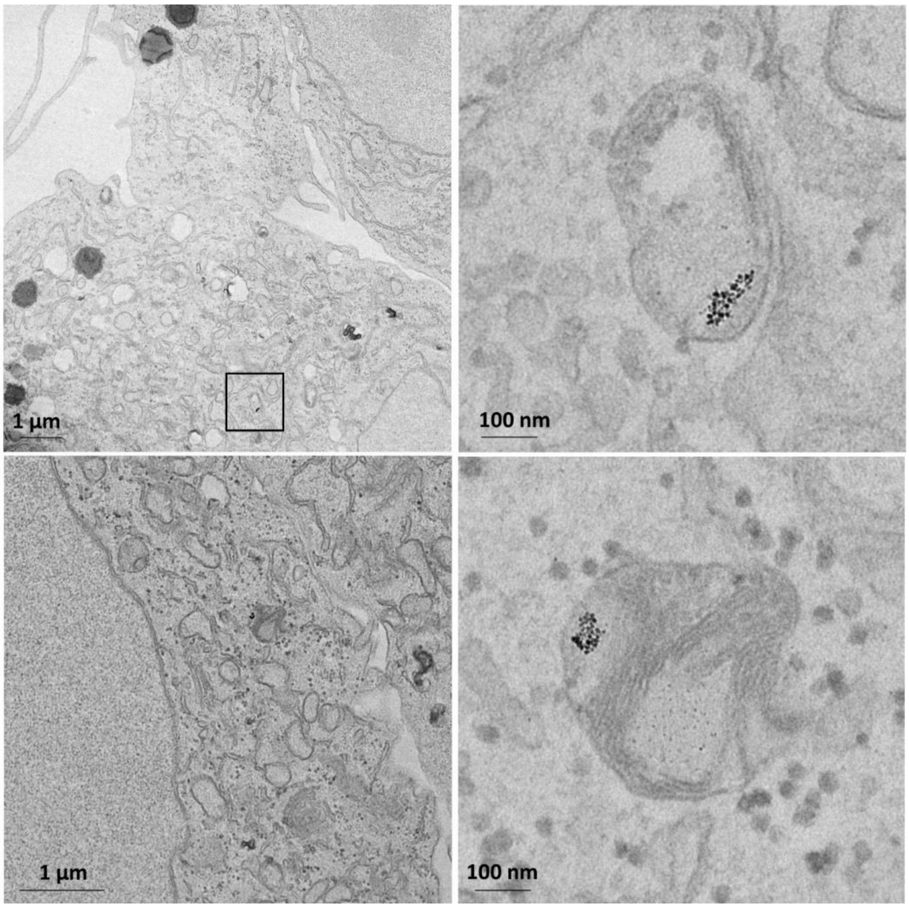

Supplementary Figure 8: TEM images of spheroids after 4 hours internalization of the nanoparticles.

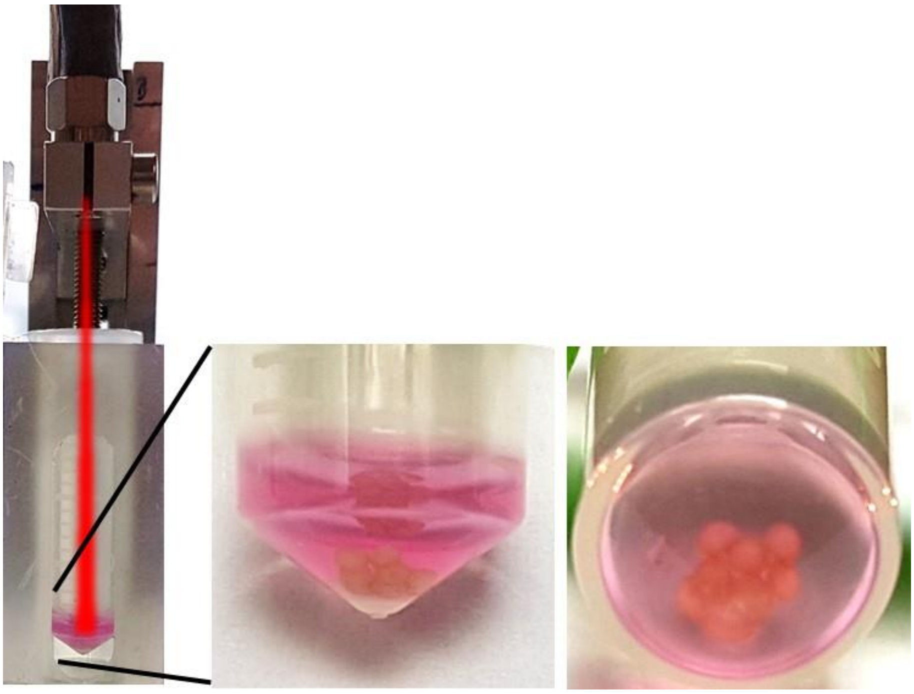

Supplementary Figure 9: Nanoparticle degradation induced by 808 nm laser application in U87 spheroids. (A) Experimental setup for photothermal application. Laser was applied at a density for 1.6 W/cm^2 for a duration of 30 min in all conditions. (B) Spheroids were placed inside a 2 mL Eppendorf tube filled with 100 µL of cell medium (*n* = 8 spheroids per condition).

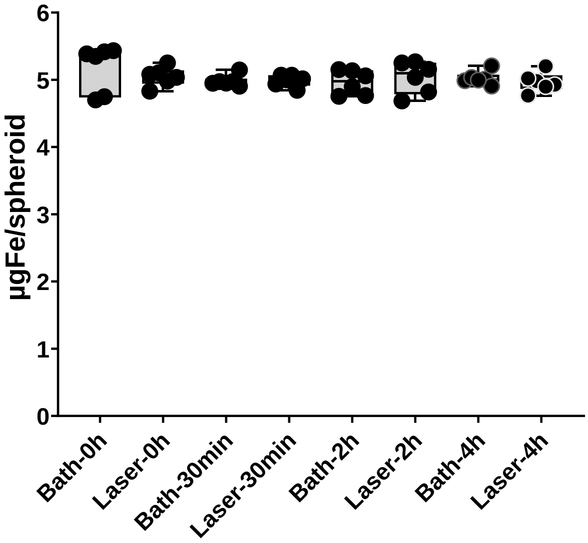

Supplementary Figure 10: Inductively coupled plasma atomic emission spectroscopy iron quantification after photothermal treatment at different spheroid maturation times and compared to the control heated in bath. There is no significant nanoparticle iron content loss highlighting that the loss of nanoparticle magnetism is due to degradation. *n* = 6 spheroids analyzed per condition. Data are presented in box plots, represented by the median, maximum and minimum data points. Unpaired two-tailed Student's t-test was used to evaluate statistical significance.

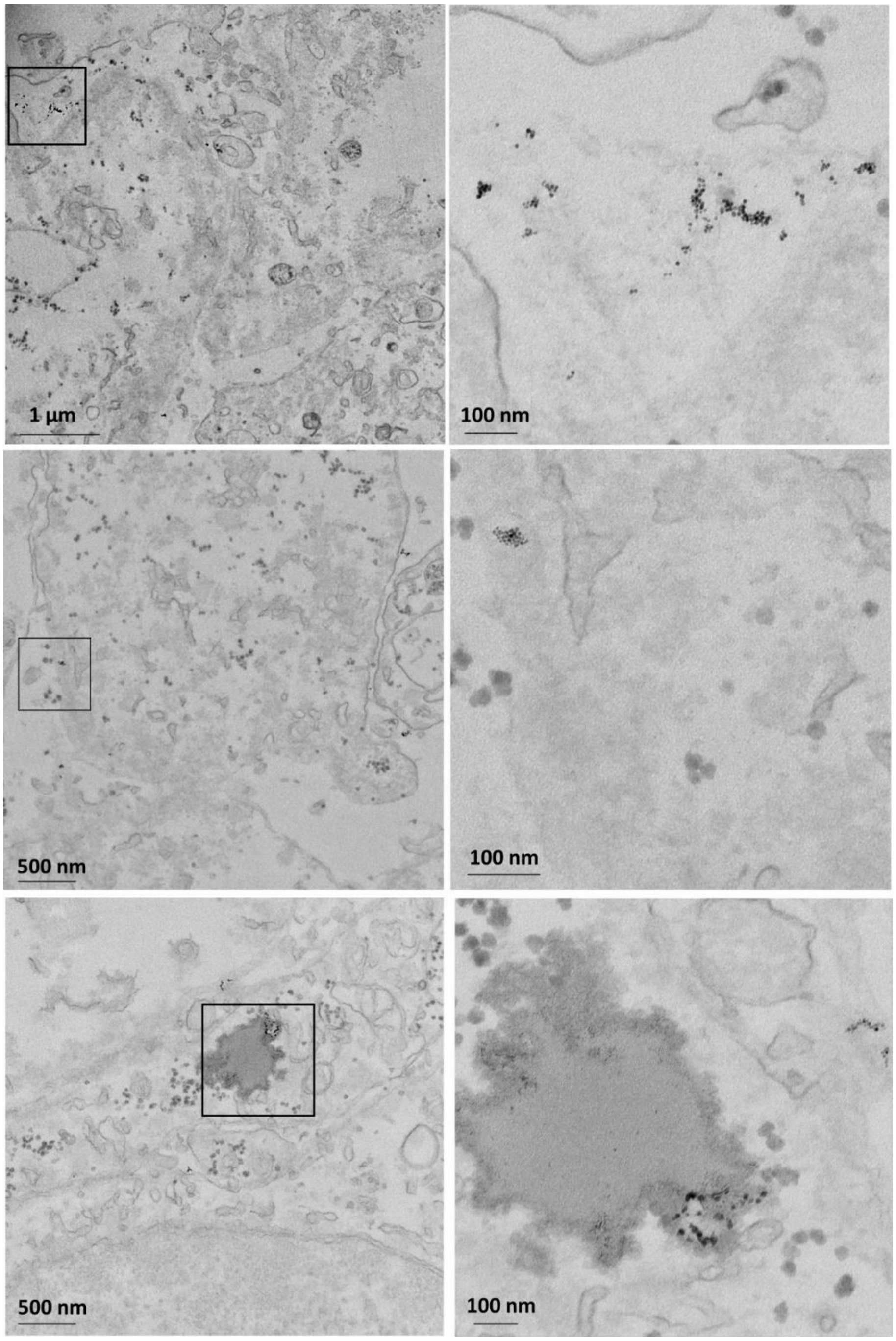

Supplementary Figure 11: TEM images of spheroids after 30 min internalization of the nanoparticles and subsequent laser exposure.

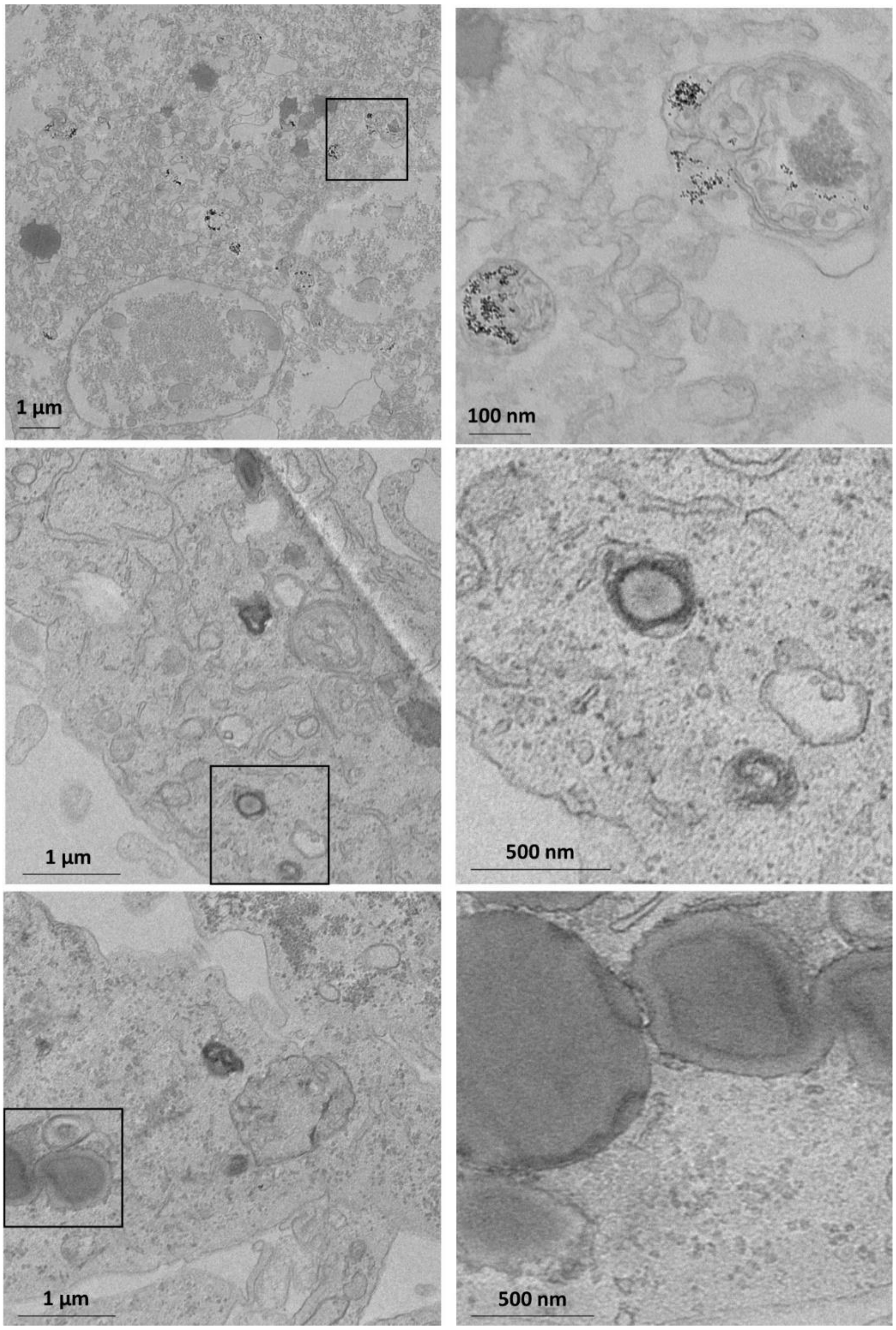

Supplementary Figure 12: TEM images of spheroids after 4 hours internalization of the nanoparticles and subsequent laser exposure.

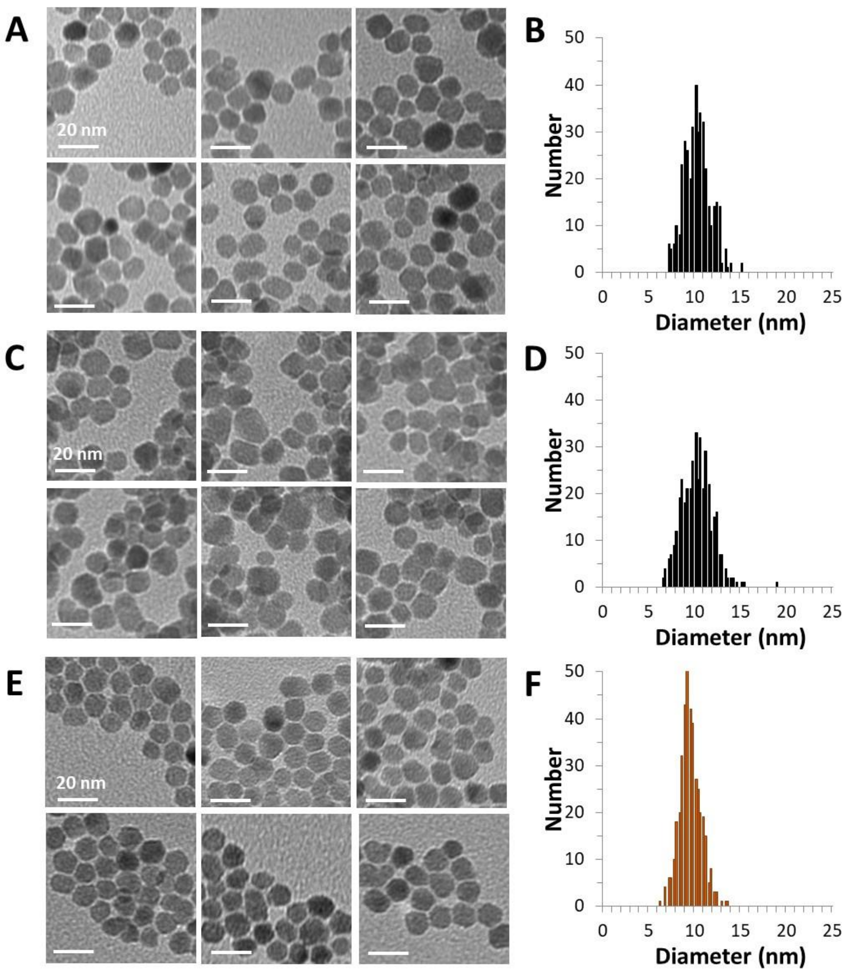

Supplementary Figure 13: TEM imaging of nanoparticles dispersed in water, without laser application (A, B), with laser application (C, D) and with laser application but at pH 4.5 supplemented with citrate (E, F). Laser was applied for 10 min at 3 W/cm² (60% degradation). Diameter distributions (B, D, F) were obtained for 400 measurement of individual nanoparticles. Average diameter in water was found to be 10.2 ± 1.4 nm and 10.2 ± 1.7 nm, without and with laser application, respectively, then decreasing significantly to 9.4 ± 1.2 nm in degrading medium with laser application.

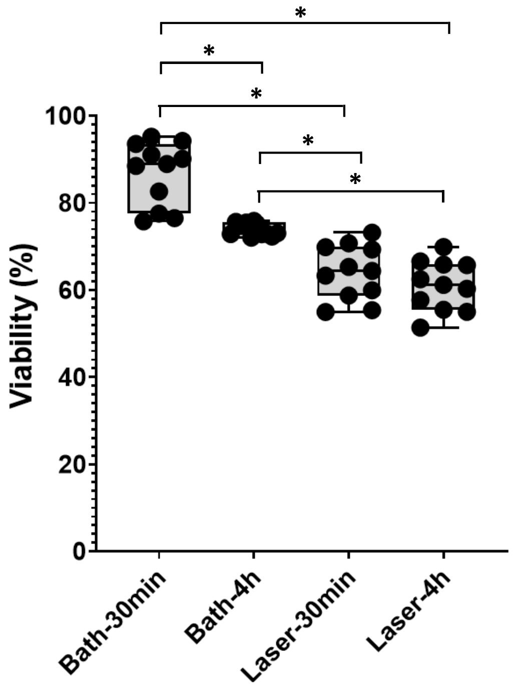

Supplementary Figure 14: Cell viability quantification in U87 tumor spheroid model after global heating and laser irradiation after an incubation of nanoparticles of 30 minutes (early endosomes) or 4 hours (late endosomes). Cell viability was assessed using a live/dead cell assay, and calculated via cell counting and fluorescence imaging. Cell viability was overall higher for controlled global heating compared to photothermal application. A significant difference in cell viability was also apparent between early and late endosomes. n = 11 spheroids analyzed per condition. Data are presented in box plots, represented by the median, maximum and minimum data points. Unpaired two-tailed Student's t-test was used to evaluate statistical significance, where $*p < 0.05$.

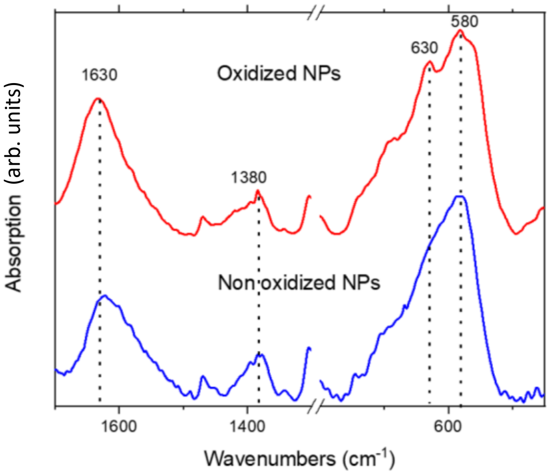

Supplementary Figure 15: FTIR of citrate coated non oxidized (NP) (blue curve) and oxidized (NPox) (red curve) nanoparticles. The FTIR spectra were recorded on a using transmission mode trough KBr pellets: Nanoparticles solution is mixed to KBr at a ratio of 5 mg FeOx for 100 mg of KBr and freeze dried overnight, and the KBr pellet is next obtained under pressure. Both NP and NPox exhibit the characteristic Fe-O vibration band within the 800-450 cm-1 region. The NP spectrum is similar to magnetite with a single peak at 580 cm^{-1} while the oxidized sample NPox exhibit a peak at 630 cm^{-1}, signature of maghemite with the formation of a spinel phase.

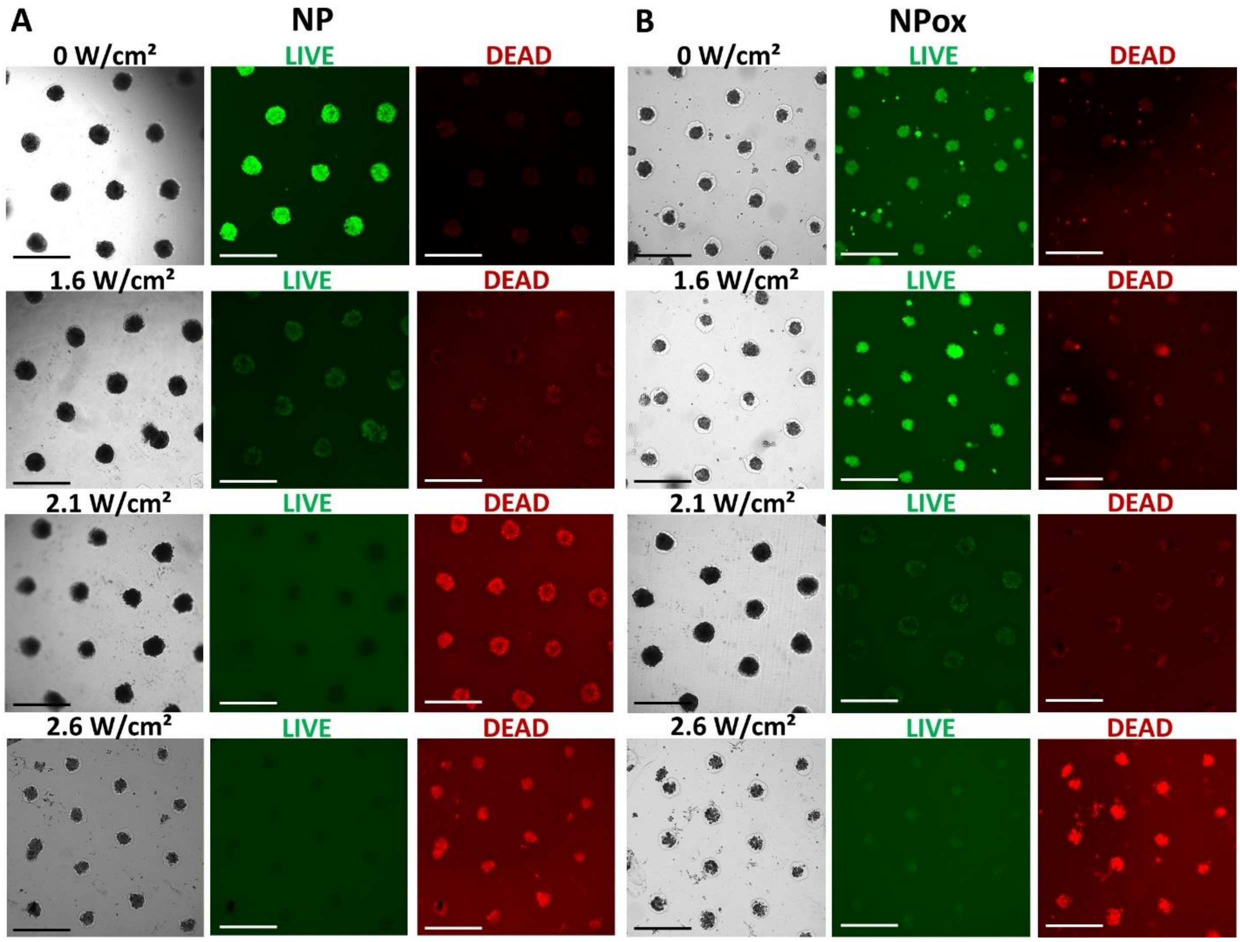

Supplementary Figure 16: Microwell patterns with Live Dead staining after laser exposure at different laser power density (W/cm²), for non-oxidized nanoparticles (NPs, A) and oxidized ones (NPox, B). Scale bars = 500 µm.

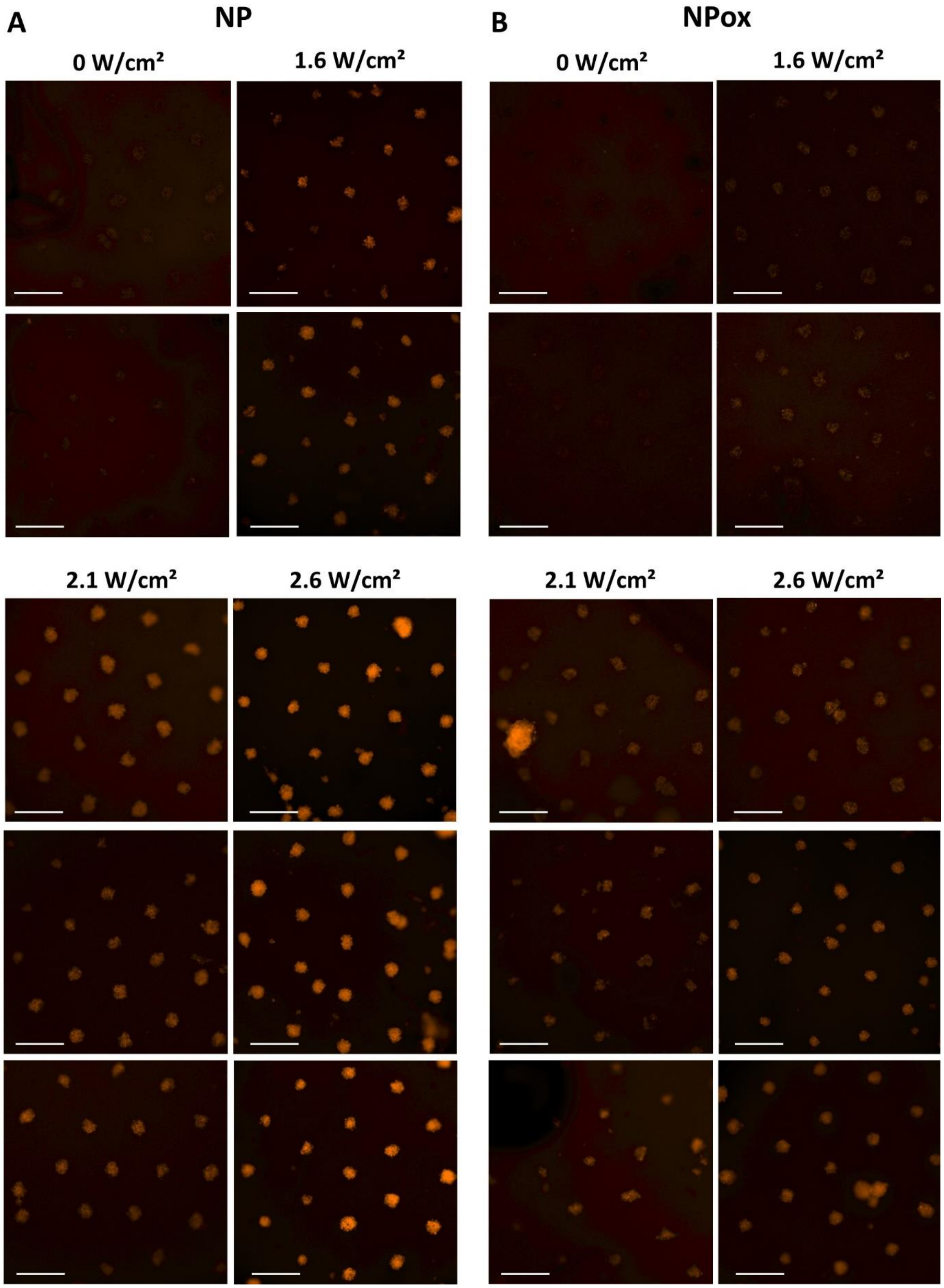

Supplementary Figure 17: Microwell patterns with FerroOrange staining after laser exposure at different laser power densities (W/cm²), for non-oxidized nanoparticles (NPs, A) and oxidized ones (NPox, B). Scale bars = 500 µm.

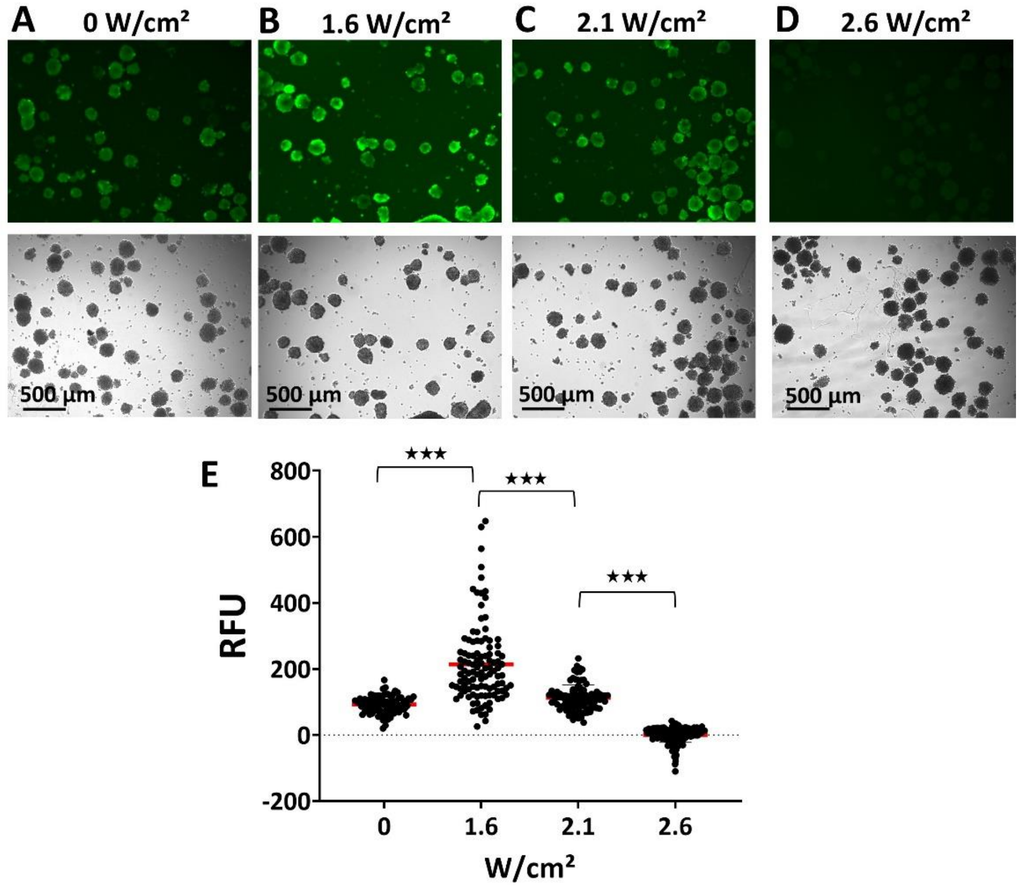

Supplementary Figure 18: ROS generation in cells. Briefly, spheroids were formed in microwells (using a 6-well plate stamp to generate 500 spheroids per well) from an initial seeding density of 500 cells labelled with nanoparticles, and matured over 48 hours. They were then transferred to a 96-well plate (100 per well) and exposed to laser with the same protocol as introduced in the Methods, at power densities of 1.6, 2.1, and 2.6 W/cm² for a duration of 5 min. 1 hour after exposure, the spheroids were washed with Hank's Balanced Salt Solution (HBSS, Thermo Fisher Scientific) and incubated with 100 µL of Highly Sensitive DCFH-DA reconstituted at 1:1000 in Loading Buffer (ROS Assay Kit – Highly Sensitive DCFH-DA-, R252, Dojindo) for 30 minutes at 37°C. The spheroids were then washed again in HBSS and observed in a EnSight® Multimode Microplate Reader (Perkin Elmer) using the green fluorescent filter (excitation: 488 nm, emission: 500-550 nm). Images show the ROS signal in green fluorescence, with the corresponding bright field for (A) control spheroids, as well as the laser power densities of (B) 1.6, (C) 2.1 and (D) 2.6 W/cm². An important generation of ROS was found for the 1.6 W/cm² condition, that decreased for 2.1 W/cm², and was almost zero for 2.6 W/cm². (E) Quantification of the relative fluorescent signal at single spheroid level (n = 100) confirms the massive ROS generation at low laser power decreasing with laser power. n values represent the number of analyzed spheroids per condition. Unpaired two-tailed Student's t-test was used to evaluate statistical significance, where ***p < 0.0001.

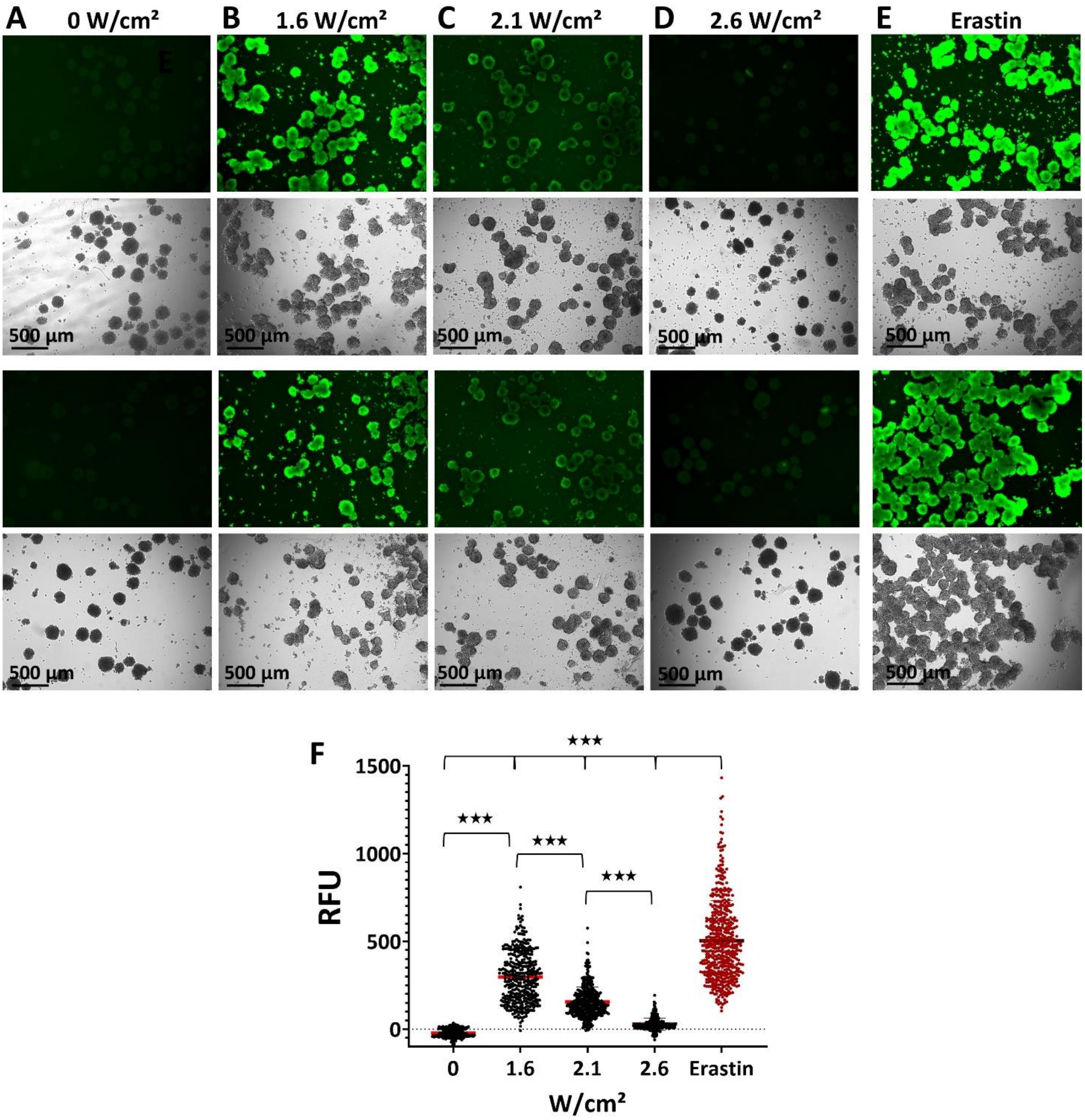

Supplementary Figure 19: Lipid peroxidation in cells. Similarly to the previous ROS analysis setup, spheroids were irradiated at increasing laser power densities of 1.6, 2.1 and 2.6 W/cm^2 for a duration of 5 minutes. 3 hours later, the spheroids were washed with HBSS and then incubated with the fluorescent dye LiperFluo (L248, Dojindo) at 5 µmol/L for 30 minutes at 37°C. Erastin (Sigma-Aldrich, E7781) was used as positive control to induce ferroptosis. Spheroids were then treated with Erastin (5 µM) for 48 hours before incubation with LiperFluo. All spheroids were washed with HBSS before imaging in a EnSight® Multimode Microplate Reader using the green fluorescent filter (excitation: 488 nm, emission: 500-550 nm). (A) Typical fluorescent and bright field images of (A) control, and the three laser conditions (B) 1.6, (C) 2.1 and (D) 2.6 W/cm², as well as (E) the Erastin condition. Lipid peroxidase presence is high for the lower power density condition (yet lower than with Erastin administration), whereas it is almost non-existent for the higher power density. (F) Quantitative analyses of single spheroids relative fluorescence intensity (*n* = 300). *n* values represent the number of analyzed

spheroids per condition. Unpaired two-tailed Student's t-test was used to evaluate statistical significance, where ***$p < 0.0001$.

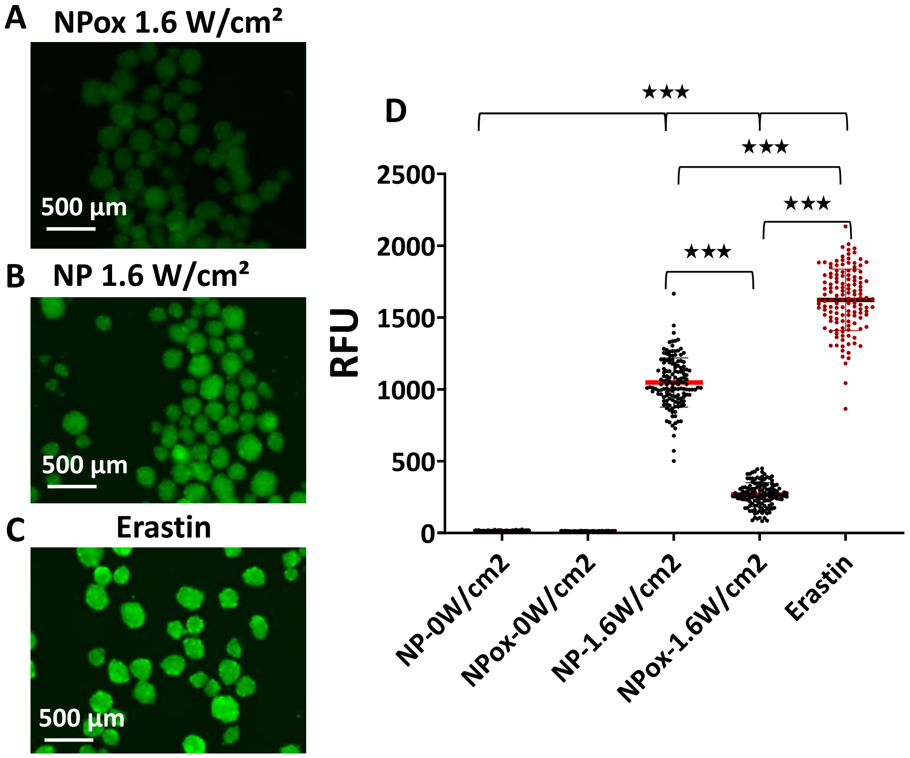

Supplementary Figure 20: Similarly to previous settings describes in supplementary Figures 18 and 19, spheroids were generated from cells labelled with nanoparticles. Herein, the two types of nanoparticles were investigated, oxidized (NPox) and non-oxidized (NP) ones. Exactly as described in Figure 19, spheroids were irradiated at laser power density of 1.6 W/cm², or submitted to Erastin treatment (5 µM), and incubated with the fluorescent dye LiperFluo. Typical fluorescent images of (A) NPox and (B) NP spheroids laser irradiated, and upon (C) Erastin treatment, are shown. Lipid peroxidase presence is high for the lower power density condition (yet lower than with Erastin administration), whereas it is almost non-existent for the higher power density. (D) Quantitative analyses of single spheroids relative fluorescence intensity (n = 100), evidencing that lipid peroxidase presence is much lowered with the use of oxidized nanoparticles NPox. n values represent the number of analyzed spheroids per condition. Unpaired two-tailed Student's t-test was used to evaluate statistical significance, where ***$p < 0.0001$.

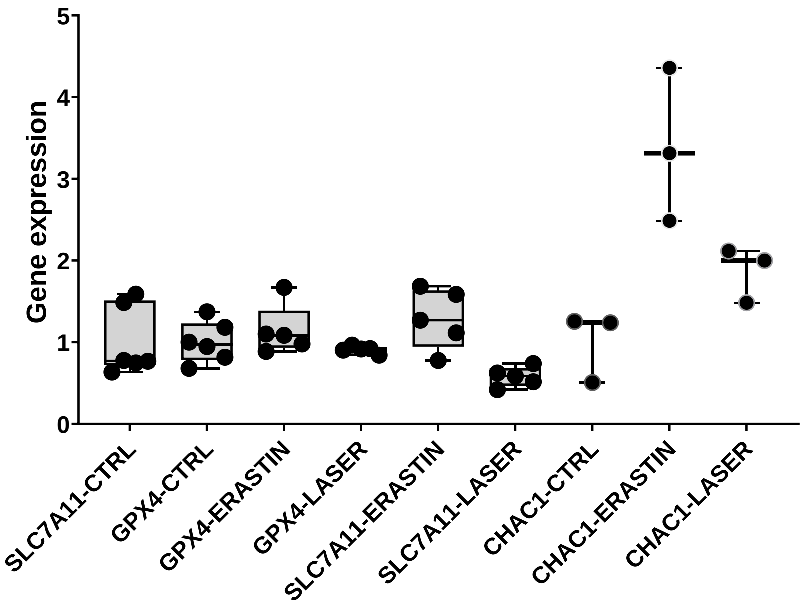

Supplementary Figure 21: Gene expression of ferroptosis-related genes GPX4, SLC7A11 and CHAC1 in U87 spheroids incubated with the ferroptosis agent Erastin (Sigma-Aldrich, E7781, 10 µM for 24 hours) or under laser irradiation (1.6 W/cm^2 for 5 minutes). Briefly, total RNA was isolated using the NucleoSpin RNA kit (Machery-Nagel), following the vendor's instructions. cDNA was synthesized using the SuperScript II Reverse Transcriptase kit (Thermo Fisher Scientific), following the vendor's instructions. Total RNA and cDNA from the previous steps were quantified using a NanoDrop OneC UV-Vis Spectrophotometer (Thermo Fisher Scientific). Real-time PCR analysis was then carried out using the SYBR Green qPCR Master Mix and the QuantStudio™ 3 Real-Time PCR System (Thermo Fisher Scientific). Results show the gene expression normalized first to the mRNA of reference transcript 60s acidic ribosomal protein P0 (RPLP0), and then normalized to controls (cells labelled with nanoparticles but without neither laser exposure nor Erastin administration). Primer sequences of the three analyzed genes are listed in the table below:

**Target gene**	**Accession number in GenBank**	**Forward primer (5' --> 3')**	**Reverse primer (5' --> 3')**
GPX4	NM_002085.4	ACAAGAACGGCTGCGTGGTGAA	GCCACACACTTGTGGAGCTAGA
SLC7A11	NM_014331.4	TCCTGCTTTGGCTCCATGAACG	AGAGGAGTGTGCTTGCGGACAT
CHAC1	NM_024111	GTGGTGACGCTCCTTGAAGATC	GAAGGTGACCTCCTTGGTATCG

The glutathione peroxidase GPX4 is an enzyme that has glutathione (GSH) for main cofactor and reduces lipid peroxides, thus protecting cells against membrane lipid peroxidation. Herein, it is not downregulated for both conditions. The Solute Carrier Family 7 Member 11 (SLC7A11) gene encodes for a cystine/glutamate antiporter. Inside the cell, cystine is converted in cysteine that is utilized to synthesize GSH. The decreased expression of SLC7A11 for the laser condition could therefore be associated with reduced intracellular GSH and the promotion of ferroptosis. ChaC Glutathione Specific Gamma-Glutamylcyclotransferase 1 (CHAC1) is one of the main upregulated genes upon erastin treatment. It mediates GSH degradation and consequently is a promoter of ferroptosis. It is upregulated for both Erastin and laser treatment. $n \geq 3$, where n values represent the number of independent replicas. Data are presented in box plots, represented by the median, maximum and minimum data points.

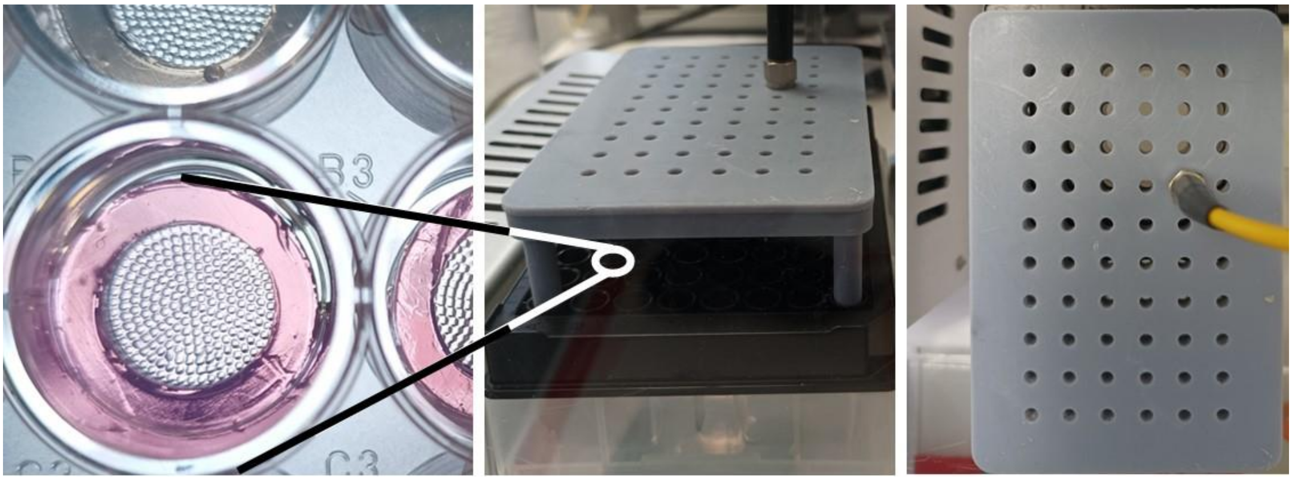

Supplementary Figure 22: Laser application on 150 µm U87 spheroids grown in agarose micro-wells. Left panel shows the custom-made agarose stamp with the microwells inside a well of a 96-well plate. Middle and right panels show the positioning of the well plate relative with the laser holder and the laser line being held in place within the holder, respectively.
